# Pediatric Evans Syndrome as a Multisystem Immune Disorder: A 13-Year Longitudinal Experience from a Single Academic Center

**DOI:** 10.3390/pediatric18020034

**Published:** 2026-03-03

**Authors:** Dimitrios Karamitsos, Ioanna Paraskevi Papandrea, Nikoletta Rokidi, Ioanna Saougou, Chrysoula Kosmeri, Alexandros Makis

**Affiliations:** 1 Faculty of Medicine, University of Ioannina, 45500 Ioannina, Greece; dimikar8@gmail.com (D.K.); ioannapapandrea@gmail.com (I.P.P.); nicolerokidi@gmail.com (N.R.); 2 Department of Pediatrics, University Hospital of Ioannina, 45500 Ioannina, Greece; ioannasaougou@gmail.com (I.S.); chrisa.kosmeri@gmail.com (C.K.); 3 Child Health Department, Faculty of Medicine, University of Ioannina, 45500 Ioannina, Greece

**Keywords:** Evans syndrome, pediatric autoimmune cytopenia, immune dysregulation, autoimmune neutropenia, inborn errors of immunity, TNFAIP3

## Abstract

**Background**: Pediatric-onset Evans syndrome (pES) is a rare autoimmune disorder defined by the coexistence or sequential development of immune thrombocytopenia (ITP) and autoimmune hemolytic anemia (AIHA), frequently accompanied by autoimmune neutropenia (AIN) and characterized by a relapsing, multilineage course. Increasing evidence suggests that pES may represent a broader immune dysregulation phenotype rather than an isolated hematologic disorder. **Methods**: We conducted a retrospective, single-center study of children diagnosed with pES and followed for up to 13 years at a tertiary referral center. Clinical data regarding hematologic evolution, extra-hematological immunopathological manifestations, treatment requirements, infectious complications, and genetic findings were analyzed descriptively. **Results**: Six children (4 males) were included, with a median age at first cytopenia of 7 years (range 3–15) and a median follow-up of 8 years (range 1–13). ITP preceded AIHA in 3/6 patients (50%), one patient (16.7%) developed AIHA first, and two (33.3%) showed partial or evolving multilineage disease with DAT positivity prior to overt hemolysis. AIN occurred in 3/6 patients (50%). Extra-hematological immunopathological manifestations occurred in 5/6 patients (83.3%), with two (33.3%) developing more than one. Second-line therapy was required in 3/6 patients (50%). Infectious episodes occurred in 83.3% of patients, predominantly viral or mild bacterial infections, with no life-threatening events. Whole-exome sequencing performed in three patients identified a heterozygous TNFAIP3 variant of uncertain significance in one case; no pathogenic variants were detected. **Conclusions**: pES demonstrates clinical heterogeneity, frequent multilineage cytopenia, and substantial extra-hematological immune involvement. Multisystem manifestations may be associated with increased treatment burden. Long-term multidisciplinary monitoring and cautious interpretation of genetic findings are essential for individualized pediatric care.

## 1. Introduction

Evans syndrome (ES) is a rare autoimmune disorder characterized by the coexistence or sequential development of immune thrombocytopenia (ITP) and autoimmune hemolytic anemia (AIHA). Autoimmune neutropenia (AIN) may also occur and is included in some clinical definitions and pediatric series. First described in 1951, ES has traditionally been considered a chronic and frequently relapsing hematologic condition with variable prognosis [1]. However, accumulating pediatric evidence suggests that pediatric-onset Evans syndrome (pES) represents a heterogeneous clinical entity extending beyond isolated autoimmune cytopenias.

pES is uncommon, accounting for a small proportion of autoimmune cytopenias in childhood, with an estimated incidence of approximately 0.5–1.2 cases per million children per year, although precise epidemiological data remain limited due to the rarity of the disease and variability in diagnostic criteria. Compared with adult-onset disease, pES more frequently follows a relapsing course and is more often associated with underlying immune dysregulation or genetic defects [2,3]. The clinical spectrum in children ranges from mild intermittent cytopenias to severe, treatment-dependent disease requiring prolonged immunosuppression.

The pathophysiology of pES is complex and incompletely understood. Proposed mechanisms include impaired peripheral immune tolerance, dysregulated T-cell activation, defective regulatory T-cell function, abnormal B-cell maturation, and enhanced autoantibody production. Altered cytokine signaling and disturbances in immune checkpoint pathways have also been implicated. Increasing evidence suggests that, in a subset of patients, ES represents a phenotypic manifestation of underlying inborn errors of immunity (IEI), particularly disorders affecting immune regulation and lymphocyte homeostasis [4]. This immunobiological heterogeneity likely explains the variability in clinical severity, organ involvement, and treatment response observed among pediatric patients.

In children, ES is often marked by recurrent cytopenias, prolonged corticosteroid exposure, and a frequent need for second-line immunomodulatory therapies. Importantly, several large cohort studies have demonstrated that a substantial proportion of pediatric patients develop additional immune-mediated manifestations during follow-up. The French OBS’CEREVANCE cohort, one of the largest pediatric series to date, reported frequent extra-hematological immune manifestations, including lymphoproliferation, autoimmune endocrinopathies, gastrointestinal involvement, and features suggestive of immune dysregulation syndromes [2]. Similarly, long-term follow-up data from pediatric cohorts have highlighted the evolving nature of the disease and its potential association with inborn errors of immunity [3].

Advances in genomic technologies have further reshaped the conceptual framework of pES. Whole-exome sequencing (WES) has identified pathogenic variants in immune regulatory genes such as *CTLA4*, *LRBA*, *STAT3*, and *TNFAIP3*, linking a subset of patients previously classified as “primary ES” to monogenic immune dysregulation disorders [4,5,6,7]. Nevertheless, the increasing use of genomic testing introduces interpretative challenges, as variants of uncertain significance (VUS) are frequently detected in clinically complex patients.

Despite growing recognition of the multisystem character of pES, important clinical questions remain unresolved. Long-term pediatric data from single-center cohorts are essential to clarify the temporal relationship between hematologic and extra-hematologic manifestations, quantify treatment burden, and identify phenotypic patterns that may warrant early genetic investigation. From a clinical perspective, recognizing ES as a potential multisystem immune disorder has practical implications for pediatric care. Children presenting with ES should be monitored not only for hematologic relapses but also for emerging organ-specific autoimmune or inflammatory manifestations. Early identification of immune dysregulation features may guide multidisciplinary management, inform decisions regarding immunomodulatory therapy, and prompt timely genetic evaluation. A structured, longitudinal approach is therefore essential to optimize outcomes and minimize cumulative treatment toxicity in this vulnerable population.

In this study, we present a 13-year longitudinal experience of children with pediatric-onset Evans syndrome managed at a tertiary referral center. The objectives were to (i) describe the sequence and evolution of autoimmune cytopenias; (ii) evaluate the frequency and spectrum of extra-hematological immunopathological manifestations; (iii) assess treatment escalation and therapeutic burden; and (iv) report genetic findings identified through WES in the context of clinical immune dysregulation.

## 2. Materials and Methods

### 2.1. Study Design and Setting

This retrospective observational cohort study was conducted at the Pediatric Hematology Unit of the University Hospital of Ioannina, a tertiary referral center in Epirus, Northwestern Greece. The center is the only referral unit for pediatric hematology cases in the regions of Epirus, Western Macedonia, and the Ionian Islands in Greece. The study included children diagnosed with ES between January 2011 and December 2023 and followed longitudinally. Clinical data were retrieved from electronic and paper medical records and analyzed descriptively.

### 2.2. Definition of ES

ES was defined as the coexistence or sequential development of ITP and AIHA, with or without AIN, in the absence of other identifiable causes of cytopenia at the time of diagnosis. ITP was defined as isolated thrombocytopenia (platelet count < 100 × 10^9^/L) of presumed immune origin after exclusion of secondary causes. AIHA was defined by (a) anemia appropriate for age, (b) laboratory evidence of hemolysis (elevated lactate dehydrogenase, indirect hyperbilirubinemia, reticulocytosis), and (c) a positive direct antiglobulin test (DAT). AIN was defined as an absolute neutrophil count of <1.5 × 10^9^/L with clinical and laboratory features consistent with immune-mediated neutropenia. Secondary causes of cytopenia (including malignancy, systemic lupus erythematosus, infections, and drug-induced cytopenias) were excluded based on clinical and laboratory evaluation.

### 2.3. Patient Selection and Follow-Up

All pediatric patients (<16 years at onset) meeting diagnostic criteria for ES and followed at our center during the study period were included. Follow-up duration was calculated from the first documented autoimmune cytopenia to the last recorded clinical evaluation. Relapse was defined as the recurrence of cytopenia requiring therapeutic intervention after a documented response. Chronic ITP was defined as thrombocytopenia persisting beyond 12 months from diagnosis.

### 2.4. Definition of Extra-Hematological Immunopathological Manifestations

Extra-hematological immunopathological manifestations (IMs) were defined as clinically and/or laboratory-confirmed autoimmune or inflammatory conditions involving organ systems other than the hematologic system. These included endocrine autoimmune diseases (e.g., autoimmune thyroiditis, type 1 diabetes mellitus); hepatic autoimmune involvement (e.g., autoimmune hepatitis or persistent autoimmune serology with compatible clinical features); rheumatologic manifestations (e.g., inflammatory arthritis or sacroiliitis); pulmonary immune-mediated disease; and lymphoproliferative manifestations (persistent lymphadenopathy or splenomegaly not attributable to infection or malignancy). The timing of IM relative to the first cytopenia was recorded as preceding, concurrent, or subsequent.

### 2.5. Treatment Definitions

First-line therapy included systemic corticosteroids and/or intravenous immunoglobulin (IVIG). Second-line therapy was defined as any additional immunomodulatory or immunosuppressive treatment administered due to relapsing, refractory, or treatment-dependent disease. These included rituximab, mycophenolate mofetil, eltrombopag, or other steroid-sparing agents. Treatment burden was assessed descriptively based on the need for escalation beyond first-line therapy and the number of second-line regimens administered.

### 2.6. Immunological and Genetic Evaluation

Immunologic testing was performed according to clinical indication rather than a uniform protocol throughout the study period. Assessment typically included serum immunoglobulin levels, lymphocyte subsets, and autoimmune serology, while ALPS-oriented screening was performed when lymphoproliferation or suggestive features were present. WES was not performed systematically in all patients; it was requested in those with early-onset disease, multilineage cytopenia, extra-hematological immune manifestations, or clinical suspicion of immune dysregulation. Genetic variants were classified according to American College of Medical Genetics and Genomics criteria as pathogenic, likely pathogenic, or VUS. Only variants in genes associated with immune regulation or inborn errors of immunity were considered clinically relevant.

### 2.7. Statistical Analysis

Data were analyzed using descriptive statistics. Continuous variables are presented as median and range. Categorical variables are expressed as absolute numbers and percentages. Given the small sample size, no inferential statistical analyses were performed.

To provide a regional epidemiologic perspective, we estimated a crude service-based annual case rate of pES syndrome within our tertiary referral catchment area (Epirus, Western Macedonia, and the Ionian Islands) over the accrual period January 2011–December 2023. Pediatric population denominators were obtained from the Hellenic Statistical Authority (ELSTAT) 2021 Population and Housing Census [8]. Because census data are reported in age bands of 0–9 and 10–19 years, the 0–16 pediatric population was approximated as the sum of the 0–9 age group and 7/10 of the 10–19 age group, assuming an approximately uniform age distribution within that band. The annual case rate was calculated as the number of newly identified pediatric-onset Evans syndrome cases managed at our institution during the study period divided by the estimated pediatric population aged 0–16 years and the number of calendar years, expressed per 10^6^ children per year. Given the small number of observed events, 95% confidence intervals for the estimated rate were calculated using the exact Poisson method based on the observed case count and corresponding person-years at risk. The resulting estimate represents a crude service-based catchment rate rather than a population-based registry incidence.

### 2.8. Ethical Considerations

The study was conducted in accordance with the Declaration of Helsinki and approved by the Institutional Review Board of the University Hospital of Ioannina (approval number: 512/15-10-2015). Informed consent was not obtained from parents or legal guardians due to the retrospective nature of the study. Data were analyzed in anonymized form.

## 3. Results

The characteristics of the patients are shown in Table 1 and Table 2.

Six children with pediatric-onset Evans syndrome were included. Based on ELSTAT 2021 census data for the region of Epirus in Greece [8], the estimated pediatric population aged 0–16 years was approximately 116,246 children after adjustment of age bands. During the accrual period 2011–2023, six children with pediatric-onset Evans syndrome were managed at our tertiary center. This corresponds to a crude service-based annual case rate of approximately 4.0 cases per 10^6^ children aged 0–16 years per year (95% exact Poisson CI: 1.46–8.64) within our referral catchment area.

The cohort comprised four males and two females. The median age at first cytopenia was 7 years (range 3–15 years). The median follow-up duration from first cytopenia was 8 years (range 1–13 years), allowing longitudinal evaluation of disease progression and immune-related complications.

The sequence of cytopenias varied considerably. ITP preceded AIHA in three patients (50%). One patient (16.7%) presented initially with warm AIHA and developed ITP four years later. Two patients (33.3%) presented with simultaneous or evolving multilineage immune abnormalities, including DAT positivity and/or autoimmune neutropenia preceding overt hemolysis or sustained thrombocytopenia. AIN was documented in three patients (50%) and was supported by positive anti-HNA antibodies in all three. One patient demonstrated increased double-negative T cells but did not fulfill the criteria for autoimmune lymphoproliferative syndrome. All patients experienced relapsing disease, although severity and treatment requirements differed significantly.

All six patients received first-line therapy with corticosteroids and/or IVIG. Second-line therapy was required in three patients (50%). These included rituximab, mycophenolate mofetil, azathioprine, and eltrombopag. The median number of second-line regimens among treated patients was 2 (range 1–3). Notably, all patients requiring treatment escalation had at least one extra-hematological IM. The remaining three patients achieved disease control without sustained immunosuppressive escalation. Treatment decisions were driven by the hematological disease course.

Infectious episodes were documented in five of six patients (83.3%) during follow-up. Three patients experienced viral infections, including influenza, COVID-19 (without complications), and respiratory syncytial virus (RSV). Two patients developed clinically significant bacterial infections: one patient had bacterial pneumonia, and another experienced one episode of bacterial parotitis and one episode of urinary tract infection. One patient had no documented infectious complications. No life-threatening infections, intensive care admissions, or infection-related deaths were recorded during the study period.

**Figure 1:**
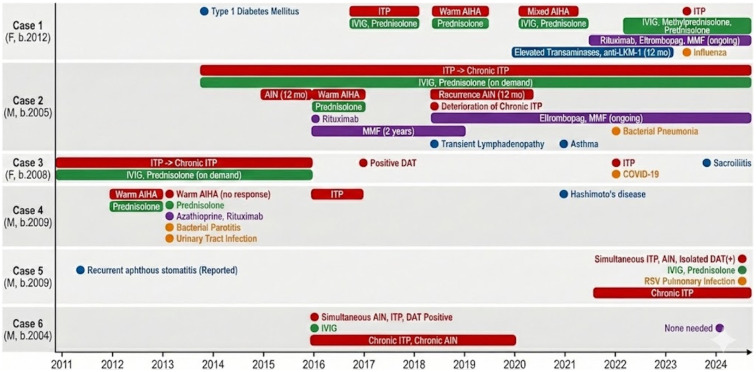
Clinical timelines of pediatric Evans syndrome. This figure presents a detailed longitudinal analysis of six pediatric patients diagnosed with Evans syndrome, spanning from 2011 to 2024. Each timeline illustrates the clinical progression, therapeutic interventions, and associated comorbidities for each case. Red = autoimmune hematologic manifestations; blue = extra-hematological immunopathological manifestations; green = first-line therapy; purple = second-line therapy; orange = infections.

Five of the six patients (83.3%) developed at least one extra-hematological IM. Two patients (33.3%) developed more than one. The spectrum included type 1 diabetes mellitus (n = 1); autoimmune thyroiditis (n = 1); suspected autoimmune hepatitis based on anti-LKM1 positivity and transient transaminase elevation (n = 1); sacroiliitis (n = 1); transient lymphoproliferation with florid follicular hyperplasia (n = 1); asthma with normal high-resolution chest CT (n = 1); and recurrent aphthous stomatitis (n = 1). In four of the five IM-positive patients (80%), extra-hematological manifestations developed after the onset of cytopenias. Extra-hematological IMs were managed in parallel. The clinical timelines are shown in Figure 1.

WES was performed in three patients (50%). In one child, a heterozygous missense variant in the *TNFAIP3* gene (*c.1733C>T*; *p.Ser578Phe*) was identified. Trio segregation analysis confirmed maternal inheritance of the heterozygous *TNFAIP3* variant, which was absent in the father; the mother was clinically asymptomatic, with no history of autoimmune or autoinflammatory disease. Variants in *TNFAIP3* are associated with autosomal dominant autoinflammatory syndromes within the A20 haploinsufficiency spectrum; however, the detected variant has not been previously reported as pathogenic and is currently classified as a VUS. Functional validation was not available, and cytokine profiling was not performed. No pathogenic or likely pathogenic variants were detected in the remaining tested patients. Therefore, while one patient carried a potentially relevant immune gene variant, no molecularly confirmed inborn error of immunity was established in this cohort.

Overall, this 13-year single-center experience demonstrates heterogeneous hematologic evolution, frequent multilineage cytopenia, substantial extra-hematological immune involvement, and variable treatment burden among children with ES.

## 4. Discussion

This 13-year single-center cohort reinforces the evolving concept that pES is not merely a refractory autoimmune cytopenia but frequently represents a broader immune dysregulation phenotype. Although originally described as the coexistence of AIHA and ITP [1], subsequent pediatric studies have demonstrated that ES in children often follows a dynamic and multisystem course [2,3,9].

The heterogeneity of hematologic presentation observed in our cohort mirrors findings from larger multicenter studies. In the French OBS’CEREVANCE cohort, sequential and simultaneous presentations were both common, and nearly half of the patients developed multilineage cytopenia over time [2]. Similarly, long-term pediatric follow-up data indicate that initial isolated autoimmune cytopenias may evolve into full ES months to years later [3,10]. The median two-year interval between cytopenias in our cohort aligns with previous observations and emphasizes the need for prolonged monitoring in children initially diagnosed with single-lineage autoimmunity.

Multilineage involvement, including autoimmune neutropenia in 50% of our patients, further supports the concept of systemic immune activation. Comparable frequencies have been reported in pediatric series ranging from 30 to 60% [2,11]. Mechanistically, impaired peripheral tolerance, dysregulated T-cell activation, and defective regulatory pathways have been implicated in the pathogenesis of Evans syndrome [4,12]. Such mechanisms overlap with those described in immune dysregulation syndromes and primary immunodeficiencies presenting with autoimmunity [4,13].

Extra-hematological immune manifestations were observed in five of six patients (83.3%), and two patients (33.3%) developed more than one manifestation. These proportions are at the upper range of previously reported pediatric series, in which 40–70% of patients develop organ-specific autoimmune or inflammatory complications during follow-up [2,3,9]. This pattern is consistent with referral bias toward more severe and complex cases in tertiary care settings. The predominance of endocrine and inflammatory manifestations in our cohort parallels previous findings in which autoimmune thyroid disease, type 1 diabetes, and lymphoproliferation are frequently reported [2,14]. Importantly, in four of the five IM-positive patients (80%), extra-hematological manifestations developed after the onset of cytopenias, supporting a progressive immune dysregulation model rather than a static autoimmune condition.

The association between immune complexity and treatment burden is particularly noteworthy. Second-line therapy was required in three of six patients (50%), a proportion consistent with referral-based pediatric cohorts [2,3,15]. All three patients requiring therapeutic escalation had extra-hematological immune manifestations (100%), reinforcing the clinical association between multisystem immune involvement and increased disease severity. The second-line agents used in our cohort included rituximab (n = 2), mycophenolate mofetil (n = 2), azathioprine (n = 1), and eltrombopag (n = 1), with a median of two second-line regimens per treated patient (range 1–3). This therapeutic heterogeneity reflects both disease variability and the absence of standardized escalation algorithms in pediatric Evans syndrome. Although the small sample size precludes formal predictive modeling, the consistent presence of extra-hematological manifestations among second-line-treated patients suggests that immune complexity may serve as a clinical indicator of increased therapeutic burden. Early clinical or laboratory signs that may anticipate the need for second-line treatment could be multilineage involvement or evolving DAT positivity, presence of lymphoproliferation (splenomegaly/adenopathy), abnormal immunologic markers when available, or an early relapsing course during follow-up.

Infectious morbidity is an important consideration in pES, particularly in the context of immune dysregulation and immunosuppressive therapy. In our cohort, infectious episodes were observed in 83.3% of patients; however, most infections were viral or clinically manageable bacterial events. No severe sepsis, ICU admissions, or infection-related mortality were documented. This contrasts with larger pediatric cohorts in which severe bacterial infections, including WHO grade 3–4 sepsis, have been reported in a substantial proportion of patients [2]. The relatively favorable infectious profile in our cohort, despite exposure to multiple immunomodulatory agents, may reflect careful immunosuppressive monitoring and individualized treatment escalation. At the same time, the high frequency of extra-hematological immune manifestations (83.3%) and the substantial need for second-line therapy (50%) likely reflect referral concentration to a tertiary pediatric hematology center, where patients with more complex or severe disease phenotypes are preferentially evaluated and managed.

Genetic evaluation is increasingly recognized as a crucial component of the diagnostic work-up in pES. Recent studies have identified pathogenic variants in genes involved in immune regulation—including *CTLA4*, *LRBA*, *STAT3*, *PIK3CD*, and *TNFAIP3*—in 10–65% of pediatric cohorts, depending on selection criteria and sequencing strategy [5,6,7,13,16,17]. In particular, A20 haploinsufficiency due to *TNFAIP3* mutations has been associated with early-onset autoimmunity, autoinflammatory features, immune dysregulation, and variable Behçet-like features [7,18]. However, the detection of VUS remains common, particularly in small or unselected cohorts [17,19]. In our study, the identified *TNFAIP3* variant is currently classified as a VUS; functional validation was not available, and cytokine profiling was not performed; thus, the pathogenic relevance of the TNFAIP3 VUS cannot be established. The absence of phenotype in the carrier parent further necessitates cautious interpretation. This underscores the importance of cautious interpretation and, when feasible, functional validation or segregation analysis. In a multicenter cohort of 60 pediatric patients, definable systemic immune dysregulation was identified in a substantial proportion, and genetic evaluations frequently revealed variants in immunoregulatory genes, including instances of *TNFAIP3* variants associated with refractory autoimmune cytopenias [20].

Compared with some referral-based cohorts reporting second-line therapy requirements exceeding 50% [2,3,15], the proportion in our study (50%) is within the reported range but remains clinically significant. Differences in referral patterns, disease severity, and access to biologic therapies likely contribute to variability across studies. Nonetheless, half of the children in our cohort required escalation beyond first-line therapy, confirming that pediatric ES carries meaningful long-term therapeutic implications.

To provide a regional epidemiologic perspective, we estimated a crude service-based annual case rate of pES of approximately 4.0 per 10^6^ children (0–16 years) per year across Epirus, Western Macedonia, and the Ionian Islands in Greece during 2011–2023. This catchment-based estimate remains higher than registry-derived population incidence rates reported from Northern Europe. In a nationwide Danish registry study including children below 13 years of age, the incidence of pES increased over time and was reported in the range of approximately 0.5–1.2 per 10^6^ person-years [2,21]. More broadly, recent reviews emphasize the rarity of Evans syndrome, with estimated incidence figures generally in the order of a few cases per million per year, depending on age group and case ascertainment [22]. Direct comparisons should be interpreted cautiously, as our estimate is not population-based but derived from a single tertiary referral center and therefore reflects a service-based catchment rate rather than true population incidence. Referral concentration, potential cross-regional referrals, the small number of cases, and denominator approximations (age-band to 0–16 conversion) may influence the observed rate. Accordingly, this figure should be considered an approximate annual case rate within our tertiary pediatric hematology catchment area rather than a definitive regional incidence.

Several limitations must be acknowledged. The retrospective design and small cohort size limit statistical power and generalizability. Our center is the only pediatric hematology referral unit for the specified regions, and external referrals could increase the number of observed cases. We therefore emphasize that this estimate is not a population-based incidence and should be interpreted with caution. Another limitation is that the non-systematic immunologic testing across the cohort and over time may have limited our ability to identify a defined IEI. Moreover, genetic testing was not performed in all patients, potentially underestimating the prevalence of monogenic immune dysregulation. However, the strength of the study lies in its prolonged follow-up and detailed phenotypic characterization within a real-world tertiary pediatric setting.

The findings of this study confirm the need to approach pES as a potentially multisystem immune disorder. Regular assessment for endocrine, hepatic, pulmonary, and rheumatologic manifestations should be incorporated into long-term follow-up protocols. The coexistence of multiple immune manifestations may identify patients at risk for increased treatment burden. Genetic testing should be considered in children with complex or early-onset disease, but interpretation of variants—particularly those classified as uncertain—requires multidisciplinary expertise and correlation with clinical phenotype [4,5,6,7,16,17]. Early recognition of immune dysregulation may allow for more targeted therapeutic strategies and reduce cumulative corticosteroid exposure.

## 5. Conclusions

In conclusion, pES should be regarded as a dynamic and heterogeneous immune disorder rather than solely a hematologic diagnosis. The high prevalence of multisystem immune involvement and the substantial need for treatment escalation highlight the need for longitudinal, multidisciplinary care and thoughtful integration of genomic data into clinical decision-making.

## Figures and Tables

**Table 1:** Clinical characteristics of the six pediatric patients with Evans syndrome.

Patient	Sex	Age at First Cytopenia	First Cytopenia	AIN	Extra-Hematological IM	IM > 1	WES	Genetic Finding	Second-Line
1	F	5	ITP	No	T1DM; autoimmune hepatitis	Yes	Yes	Negative	Yes
2	M	9	ITP	Yes	Transient lymphadenopathy (florid follicular hyperplasia); asthma (HRCT normal)	Yes	Yes	Negative	Yes
3	F	3	ITP	No	Sacroiliitis	No	Yes	*TNFAIP3* VUS	No
4	M	3	AIHA	No	Hashimoto thyroiditis	No	No	—	Yes
5	M	15	ITP + AIN (DAT+ without hemolysis)	Yes	Aphthous stomatitis	No	No	—	No
6	M	14	ITP + AIN with persistent DAT positivity (no hemolysis)	Yes	None	No	No	—	No

Abbreviations: ITP, immune thrombocytopenia; AIHA, autoimmune hemolytic anemia; AIN, autoimmune neutropenia; IM, immunopathological manifestation; WES, whole-exome sequencing; VUS, variant of uncertain significance; HRCT, high-resolution computed tomography.

**Table 2:** Summary of clinical and immunological characteristics of the cohort (N = 6).

Variable	Value
Demographics	
Total patients	6
Male sex	4 (66.7%)
Median age at first cytopenia, years (range)	7 (3–15)
Median follow-up, years (range)	8 (1–13)
Family history of autoimmunity/cancer	3 (50%)
Hematologic characteristics	
ITP preceding AIHA	3 (50%)
Simultaneous or evolving multilineage presentation	2 (33.3%)
AIHA preceding ITP	1 (16.7%)
Median interval to second cytopenia, years (range)	2 (0–6)
Autoimmune neutropenia (AIN)	3 (50%)
Immunopathological manifestations (IMs)	
≥1 extra-hematologic IM	5 (83.3%)
>1 IM	2 (33.3%)
IM before first cytopenia	1 (16.7%)
IM after first cytopenia	4 (66.7%)
Endocrinological IM	2 (33.3%)
Lymphoproliferation	1 (16.7%)
Gastrointestinal/hepatic (incl. autoimmune hepatitis)	1 (16.7%)
Rheumatological	1 (16.7%)
Pulmonary	1 (16.7%)
Defined PID/Secondary Evans	0 (0%)
Genetic testing	
WES performed	3 (50%)
Pathogenic IEI-related variant identified	0 (0%)
*TNFAIP3* variant of uncertain significance (VUS)	1 (16.7%)
Treatment	
First-line therapy (steroids/IVIG)	6 (100%)
Second-line therapy required	3 (50%)
Second-line agents used	Rituximab (n = 2); Mycophenolate mofetil (n = 2); Azathioprine (n = 1); Eltrombopag (n = 1)
Median number of second-line regimens (range)	2 (1–3)
IM present among second-line patients	3/3 (100%)

## Data Availability

The datasets generated during and/or analyzed during the current study are available from the corresponding author upon reasonable request.

## Abbreviations

The following abbreviations are used in this manuscript:

AIHA	autoimmune hemolytic anemia
AIN	autoimmune neutropenia
DAT	direct antiglobulin test
IEI	inborn errors of immunity
IM	immunological manifestation
ITP	immune thrombocytopenia
pES	pediatric-onset Evans syndrome
VUS	variants of uncertain significance
